# Lipid Alterations in Experimental Murine Colitis: Role of Ceramide and Imipramine for Matrix Metalloproteinase-1 Expression

**DOI:** 10.1371/journal.pone.0007197

**Published:** 2009-09-29

**Authors:** Jessica Bauer, Gerhard Liebisch, Claudia Hofmann, Christian Huy, Gerd Schmitz, Florian Obermeier, Jürgen Bock

**Affiliations:** 1 Department of Internal Medicine I, University Medical Center, Regensburg, Germany; 2 Institute for Clinical Chemistry, University Medical Center, Regensburg, Germany; Charité-Universitätsmedizin Berlin, Germany

## Abstract

**Background:**

Dietary lipids or pharmacologic modulation of lipid metabolism are potential therapeutic strategies in inflammatory bowel disease (IBD). Therefore, we analysed alterations of bioactive lipids in experimental models of colitis and examined the functional consequence of the second messenger ceramide in inflammatory pathways leading to tissue destruction.

**Methodology/Principal Findings:**

Chronic colitis was induced by dextran-sulphate-sodium (DSS) or transfer of CD4^+^CD62L^+^ cells into RAG1^−/−^-mice. Lipid content of isolated murine intestinal epithelial cells (IEC) was analysed by tandem mass spectrometry. Concentrations of MMP-1 in supernatants of Caco-2-IEC and human intestinal fibroblasts from patients with ulcerative colitis were determined by ELISA. Imipramine was used for pharmacologic inhibition of acid sphingomyelinase (ASM). Ceramide increased by 71% in chronic DSS–induced colitis and by 159% in the transfer model of colitis. Lysophosphatidylcholine (LPC) decreased by 22% in both models. No changes were detected for phosphatidylcholine. Generation of ceramide by exogenous SMase increased MMP-1-protein production of Caco-2-IEC up to 7-fold. Inhibition of ASM completely abolished the induction of MMP-1 by TNF or IL-1β in Caco-2-IEC and human intestinal fibroblasts.

**Conclusions/Significance:**

Mucosal inflammation leads to accumulation of ceramide and decrease of LPC in the intestinal epithelium. One aspect of ceramide generation is an increase of MMP-1. Induction of MMP-1 by TNF or IL-1β is completely blocked by inhibition of ASM with imipramine. Therefore, inhibition of ASM may offer a treatment strategy to reduce MMP-1 expression and tissue destruction in inflammatory conditions.

## Introduction

Many signalling molecules involved in the pathogenesis of inflammatory bowel disease (IBD) such as tumor necrosis factor (TNF) or interleukin-1 beta (IL-1β) cause alterations of the lipid composition in the cell membrane by activation of various phospholipases, sphingomyelinases and other lipid modifying enzymes [1]–[4]. Previous reports demonstrated therapeutic effects of lipid administration [5]–[7] or inhibition of lipid modifying enzymes [8] for the treatment of intestinal inflammation. Ceramide or lysophosphatidylcholine play key roles as second messengers for intracellular signalling with the potential to control inflammatory responses.

Ceramide is generated by de novo synthesis or hydrolysis of plasma membrane sphingomyelin via the action of sphingomyelinases (SMases). SMases are characterized by their optimal pH and are divided accordingly into acid, neutral and basic sphingomyelinase species. The acid sphingomyelinase (ASM) contributes to lysosomal sphingomyelin turnover and is also secreted upon cellular treatment with inflammatory stimuli [9], [10]. Ceramide has been implicated in a variety of cellular processes from cell growth, differentiation and gene transcription to cell death [11], [12]. Ceramide seems to play a central role for the pathophysiology of several common diseases [9] and is a key mediator for apoptosis, cellular invasion of bacteria and viruses, radiation- and chemotherapeutic responses, heat damage, UVA-light and ischemia-reperfusion injury [13]. Increasing evidence suggests implications of SMases in the development of colon cancer or IBD [13], [14]. Inhibition of ASM has been shown to protect from DSS-induced colitis in mice [15]. The authors suggested that the protective effect of ASM-inhibition was mediated by the suppression of cytokine production from macrophages in response to LPS. Unfortunately, the response of intestinal epithelial cells (IEC) and fibroblasts has not been investigated, although these cells may well contribute to the protective effects of ASM-inhibition.

Phosphatidylcholine (PC) is the main phospholipid component of eukaryotic cells. Instillation of PC and phosphatidylinositol have been shown to prevent acetic acid-induced colitis in the rat [5]. The formation of strictures in a rat model of colitis was also prevented by oral supplementation of polyunsaturated PC, possibly by stimulation of collagen breakdown [6]. The studies by Stremmel et al focused on the content of PC in mucus layers as the possible culprit for uncontrolled inflammation of the intestine [7], [16] which may be prevented by administration of PC. Nevertheless, in vitro studies revealed that exogenously added PC is also integrated into the cells and has anti-inflammatory properties in Caco-2 intestinal epithelial cells, especially in response to TNF [17]. Lysophosphatidylcholine (LPC) is a metabolic product of PC and displays inflammatory activity. Biosynthesis is regulated by PLA_2_ that catalyzes PC hydrolysis [18]. LPC acts as a chemotactic factor for monocytes and T cells and displays proinflammatory properties even at nanomolar concentrations [19]. Phosphatidylethanolamine (PE) and plasmalogenes (PE-pl) serve as precursors for lipoxygenases, thereby contributing to immune-regulatory activities [20].

Excessively produced Matrix Metalloproteinase-1 (MMP-1) is believed to damage the colonic mucosa in patients with ulcerative colitis [21], [22]. MMP-1, also called interstitial collagenase, belongs to a family of zinc-dependent metalloendopeptidases collectively capable of degrading essentially all extracellular matrix (ECM) components [23], [24]. Most of the MMPs are secreted as proenzymes and require proteolytic cleavage for activation [25]. Activity of MMPs is further regulated by a group of endogenous proteins, so called tissue inhibitors of metalloproteinases (TIMPs) that bind to active and alternative sites of activated MMPs [26]. The balance of activated MMPs and TIMPs is crucial to maintain tissue allostasis. Excessive production or activation of MMPs results in uncontrolled degradation of ECM [25], [27], [28]. MMP-1 is increased upon stimulation with inflammatory cytokines such as IL-1β or TNF [29], ionizing radiation [30] or UVA irradiation [31]. Expression of MMP-1 has been linked with ceramide metabolism via activation of extracellular signal-regulated and stress-activated protein kinase pathways [32], [33] but the relevance of this association for the mentioned stimuli and the involvement of endogenous SMases remain to be determined. ASM-activating cytokines and stimuli greatly overlap with the stimulatory processes which increase MMP-1. Therefore, we investigated the effects of these stimuli on MMP-1 production in IEC and intestinal fibroblasts.

To determine the occurring lipid alterations in intestinal inflammation, we quantified bioactive lipids in two murine models of chronic colitis. The increase of ceramide was linked with the secretion of MMP-1. Exogenous SMase produced high levels of MMP-1 in Caco-2 IEC and induction of MMP-1 by TNF or IL-1β was completely abrogated by inhibition of ASM with imipramine.

## Materials and Methods

### Mice

Female Balb/c (Harlan Winkelmann, Borchen, Germany) and RAG1^−/−^ mice (Taconic, USA) weighing 19–22 g were individually housed in standard polycarbonate mouse cages for at least two weeks before the start of the experiment. All mice were kept under standard laboratory conditions (12-h light/dark cycle, 22±2°C; 60±5% humidity). All experimental protocols were approved by the Committee on Animal Health and Care of the local government (AZ 621-2531.1-08/04), and conformed to international guidelines on the ethical use of animals. All efforts were made to minimize the number of animals used and their suffering.

### Cells and reagents

Caco-2 cells were maintained in Dulbecco's minimum essential medium (DMEM), supplemented with 10% fetal calf serum (FCS), 1% penicillin/streptomycin, 1% non-essential amino acids and 1% sodium pyruvate in an atmosphere containing 10% CO_2_ at 37°C. FCS was inactivated for 1h, 50°C. Isolation and cultivation of fibroblasts is described below. For quantification of MMP-1 protein by ELISA, experiments were performed without FCS. To avoid prestimulation by serum withdrawal, primary fibroblasts were maintained in medium with 0.5% FCS prior to stimulation with TNF. Sphingomyelinase from staphylococcus aureus and imipramine were purchased from Sigma-Aldrich, Taufkirchen, Germany. IL-1β, TNF and MMP-1 ELISA were purchased from R&D Systems, Wiesbaden, Germany.

### Isolation of human fibroblasts from patients

Human fibroblasts were isolated from colonic sections of patients with ulcerative colitis or sections of non-IBD patients without inflammation. Fibroblasts were isolated and cultured as previously described [34], [35]. In brief, the mucosa from surgical patients was cut into 1-mm pieces and epithelial cells were removed in Hank's Balanced Salt Solution (HBSS) without Ca^2+^ and Mg^2+^ (PAA, Cölbe, Germany) with 2mM ethylenediaminetetraacetic acid (EDTA) (Sigma-Aldrich, Taufkirchen, Germany). The remaining tissue was digested for 30 min at 37°C in phosphate-buffered saline (PBS, Gibco, Karlsruhe, Germany) containing 1 mg/ml collagenase I (Sigma-Aldrich), 0.3mg/ml DNase I (Roche, Mannheim, Germany), and 2mg/ml hyaluronidase (Sigma-Aldrich). Isolated cells were washed with DMEM containing 20% FCS and cultured in 25-cm^2^ culture flasks (Costar, Bodenheim, Germany) with DMEM containing 10% FCS, penicillin (100IU/ml), streptomycin (100 µg/ml), ciprofloxacin (8 µg/ml), gentamycin (50 µg/ml), and amphotericin B (1 µg/ml). Non-adherent cells were removed by subsequent changes of medium.

### Antibodies

The following antibodies were used for flow cytometry (FACS) analysis: Rat anti-mouse G8.8 (Ep-CAM) antibody was a kind gift by Dr. U. Strauch, Regensburg, Germany. Fluorescein (FITC) conjugated F(ab')2 fragment goat anti-rat IgG were from Jackson ImmunoResearch, Suffolk, UK.

### MMP-1 ELISA

For detection of MMP-1, fibroblasts and Caco-2 cells were seeded in 12-well plates. Experiments were performed in medium without FCS or 0.5% FCS in the case of stimulation by TNF. Cells were incubated with the indicated substances. For pharmacologic inhibition of ASM imipramine (30 µM) was used. Concentration of MMP-1 in supernatants was determined by ELISA (R&D Systems). All measurements were performed in duplicate.

### Dextran sulphate sodium (DSS) induced chronic colitis

For induction of chronic colitis mice received 3% DSS (MP Biomedicals, Illkirch, France) in drinking water for 7 days, as described previously [36]. Each cycle consisted of 3% DSS in drinking water for 7 days, followed by a 7 days interval with normal drinking water. Mice were failed to 4 cycles and were used for the experimental treatment 4 weeks after completion of the last cycle.

### CD4^+^CD62L^+^ T cell transfer model of colitis

Splenic CD4^+^CD62L^+^ T cells from Balb/c mice were isolated as described previously [37], [38]. In brief, CD4^+^CD62L^+^ T cells were purified from spleen mononuclear cells of healthy mice by CD4^+^CD62L^+^ T Cell isolation kit with immunomagnetic microbeads (Miltenyi Biotech, Bergisch Gladbach, Germany). CD4^+^CD62L^+^ T cells (0.25×10^6^) were resuspended in 200 µl of sterile phosphate buffered saline (PBS) and injected intraperitoneally in recipient RAG1^−/−^ deficient mice. Colitis activity was monitored by weight changes and histological analysis. After 6–8 weeks mice were used for experiments.

### Histological score

For the histological analyses cross sections of the colon were fixed in 4% formalin and the tissue was embedded in paraffin, sliced in sections of 2 µm thickness and stained with haematoxylin-eosin and scored as described [36], [39]. The predominant feature of microscopic inflammation in colitis is the mononuclear cell infiltration limited to the mucosa and the consecutive mucosal damage with loss of goblet cells and loss of crypts. Both features were independently graded from 0 to 4 and the mean score was noted. The total histological score represents the sum of the epithelium and infiltration score and ranges from 0 to 8.

### Affymetrix gene array

First, Caco-2 cells (6×75cm^2^ flask) were stimulated with exogenous SMase (0.1 U/ml) for 6h and 24h. After incubation, cells were isolated with RNeasy Mini Kit (Qiagen, Hilden, Germany). Gene expression profiles were determined as described before [40] using Affymetrix HGU133A and HGU133B GeneChips (Affymetrix, Santa Clara, CA), which cover 22,283 annotated human genes (U133A) and more than 33,000 human EST sequences (U133B). Caco-2 RNA stimulated with exogenous SMase (6h and 24h) was used for pooling to generate complementary RNA (cRNA). The remaining amount of RNA was used for real-time RT-PCR validation in single samples. 10 µg of pooled total RNA was converted to complementary DNA (cDNA) using a T7-oligo-d(T)_24_ primer and SuperScript reverse transcriptase (Invitrogen, Carlsbad, CA, USA). Second-strand cDNA synthesis and blunt ending was performed using T4 DNA polymerase, *Escherichia coli* DNA ligase, and T4 polynucleotide kinase. Following phenol-chloroform extraction, cDNA was used for in vitro transcription reaction using the T7 BioArray High Yield RNA Transcript Labeling Kit (Enzo Diagnostics, Farmingdale, NY, USA) to produce biotinylated cRNA. Thereafter, the labeled cRNA was purified using RNeasy Mini Kit columns (Qiagen) and fragmented by incubation at 94°C for 30 minutes. Fragmentation was checked by microcapillary electrophoresis on an Agilent 2100 bioanalyzer, and 30 µg of biotinylated cRNAs was split into 2 parts and hybridized to U133A and U133B GeneChips, respectively, for 16 hours at 45°C with constant rotation. Microarray was processed in an Affymetrix GeneChip Fluidics Station 400. Following staining with streptavidin-conjugated phycoerythrin and washing cycles, the microarrays were scanned using the GeneArray Scanner (Agilent Technologies). Expression signals for each transcript and comparisons between different samples were calculated with the Affymetrix GeneChip software MAS5.0 and Microsoft Excel (Microsoft Corp., Redmond, WA) [40].

### Tandem mass spectrometry

Intestinal epithelial cells (IECs) were isolated from mice colon as previous described [41]. After isolation, cells were washed with ice-cold PBS twice. Samples were prepared by addition of 500 µl ice-cold H_2_O with 0.2% SDS, followed by addition of 500 µl H_2_O. Lipids were quantified by electrospray ionization tandem mass spectrometry (ESI-MS/MS) in positive ion mode as described previously [42], [43]. Samples were quantified by direct flow injection analysis using the analytical setup and the data analysis algorithms described by Liebisch et al. [42]. A parent ion scan of *m/z* 184 specific for phosphocholine containing lipids was used for phosphatidylcholine, sphingomyelin and lysophosphatidylcholine. Neutral loss scans of *m/z* 141 and *m/z* 185 were used for phosphatidylethanolamine and phosphatidylserine, respectively. PE-based plasmalogens (PE-pl) were analysed by fragment ions of *m/z* 364, 380 and 382 for PE p16∶0, p18∶1 and p18∶0 species, respectively. Ceramide was analysed using N-heptadecanoyl-sphingosine as internal standard. Free cholesterol (FC) and CE species were determined after selective acetylation of FC. Quantification was achieved by calibration lines generated by addition of naturally occurring lipid species to cell homogenates.

### Statistical analysis

Data are shown using vertical Box-Whisker plots (25% and 75% values), generated in the basic module of the programs SigmaPlot/SigmaStat. Statistical analysis was performed by student's t-test or Mann-Whitney U-test, with p<0.05 considered statistically significant. Data are given as means ± SEM.

## Results

### Lipid alterations in chronic DSS–induced colitis and CD4^+^CD62L^+^ transfer model of colitis: increase of ceramide, decrease of lysophosphatidylcholine

To identify inflammation induced lipid alterations, we first analysed the occurring changes of bioactive lipids in experimental models of chronic colitis by tandem mass spectrometry.

**Figure 1:**
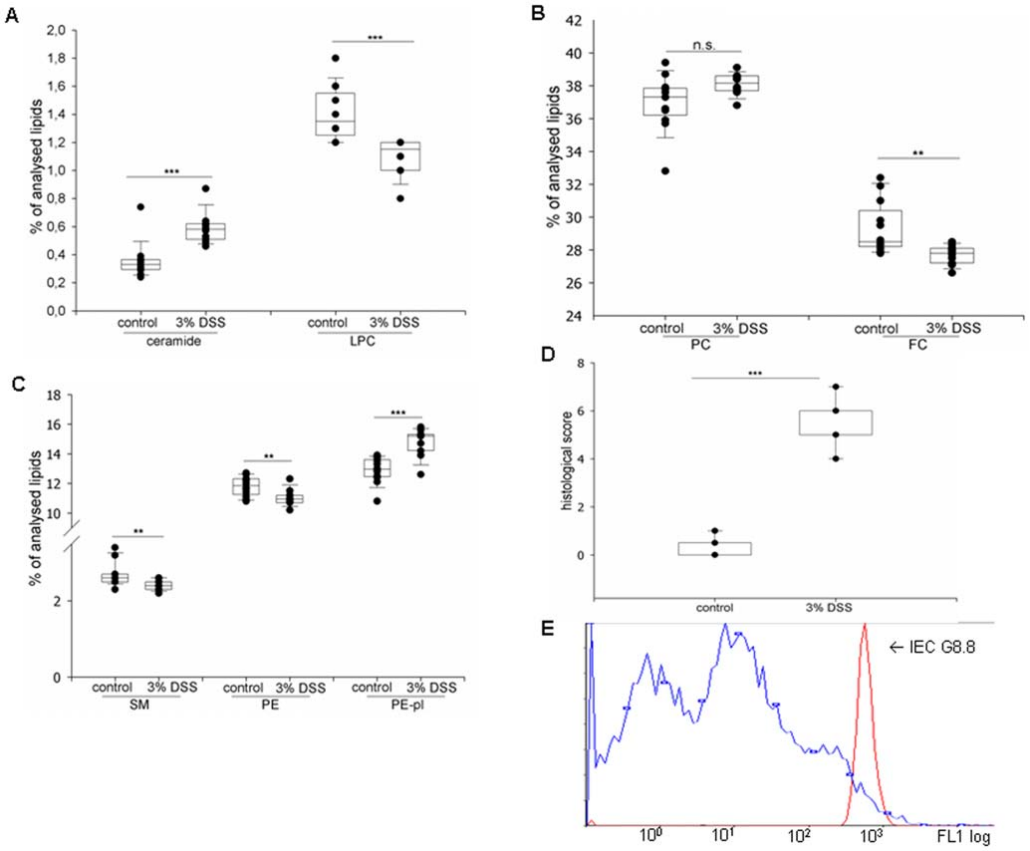
Increase of ceramide and decrease of LPC in chronic DSS-induced colitis. (A–C) Lipid analysis of isolated colonic IEC by tandem mass spectrometry (control n = 12, 3% DSS n = 10). (A) Proportion of ceramide and LPC of analysed lipids. (B) Proportion of PC and FC of analysed lipids. (C) Proportion of SM, PE and PE-pl of analysed lipids. (D) Histological score. (E) Surface expression of epithelial cell marker, determined by flow cytometry after staining of the cells with FITC-labelled anti G8.8 antibodies. (**p<0.01, ***p<0.001)

Isolated colonic IEC of mice suffering from chronic DSS-induced colitis showed the following lipid alterations when compared to control (Figure 1A–1C):

Ceramide increased by 71% (Figure 1A). In contrast, lysophosphatidylcholine (LPC) decreased by 22% (Figure 1A). Levels of phosphatidylcholine remained constant (Figure 1B). Free cholesterol (Figure 1B), Sphingomyelin (Figure 1C) and phosphatidyl-ethanolamine (Figure 1C) declined. Plasmalogenes (Figure 1C) increased. Histological score of mice with DSS-induced colitis versus control is shown in Figure 1D. To exclude contamination of IEC by other cells, isolated cells were stained with FITC-coupled anti-G8.8 antibodies, specific for epithelial cells [44]. Flow cytometry revealed that more than 93% of isolated cells were of epithelial origin (Figure 1E), indicating a good purification of IEC without relevant numbers of other mucosal cells.

**Figure 2:**
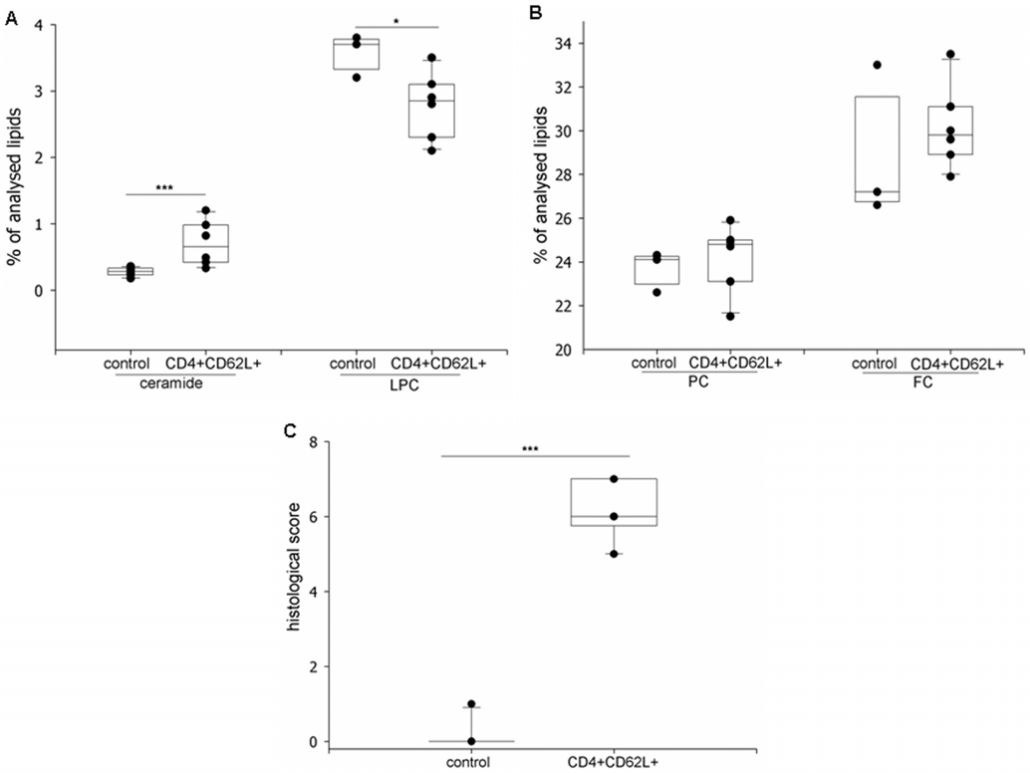
Increase of ceramide and decrease of LPC in chronic colitis by transfer of CD4^+^CD62L^+^ cells in RAG-1^−/−^ mice. (A,B) Lipid analysis of isolated colonic IEC by tandem mass spectrometry (control n = 3, CD4^+^CD62L^+^ n = 6). (A) Proportion of ceramide and LPC of analysed lipids. (B) Proportion of PC and FC of analysed lipids. (C) Histological score. (*p<0.05)

Colonic IEC from mice with chronic colitis by transfer of CD4^+^CD62L^+^ cells displayed the following changes (Figure 2A and 2B):

Ceramide increased by 159% (Figure 2A). In contrast, lysophosphatidylcholine (LPC) decreased by 22% (Figure 2A). Levels of phosphatidylcholine (Figure 2B) and free cholesterol (Figure 2B) remained constant. Histological score of mice in CD4^+^CD62L^+^ transfer model of colitis was higher compared to control mice (Figure 2C).

### Increased expression of MMP-1- and MMP-10-mRNA by exogenous SMase in Caco-2 intestinal epithelial cells (IEC)

**Figure 3:**
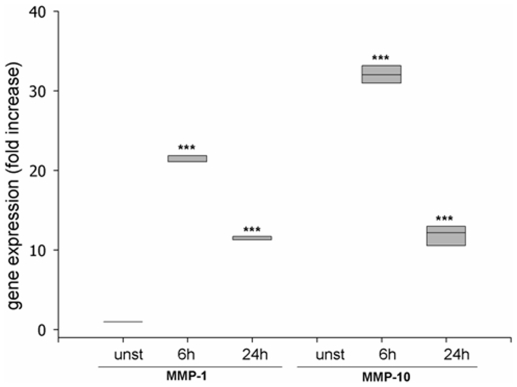
Induction of MMP-1- and MMP-10-mRNA in the intestinal epithelial cell line Caco-2 after generation of ceramide by exogenous SMase (0,1U/ml), as determined by affymetrix gene array analysis. Data are presented as relative increase after 6h and 24h in comparison to untreated control. (n = 4; ***p<0.001)

Based on the differences found in chronic colitis, we examined the effects of ceramide generation in IEC by affymetrix gene array analysis. Therefore the colorectal cancer cell line Caco-2 was incubated with exogenous SMase (0.1 U/ml) to identify ceramide induced effects possibly involved in IBD pathophysiology. The experiments revealed a fast and robust increase of MMP-1 and MMP-10 (Figure 3). The induction of MMP-10 was not further investigated.

### Exogenous Sphingomyelinase increases MMP-1 protein production in Caco-2 cells

**Figure 4:**
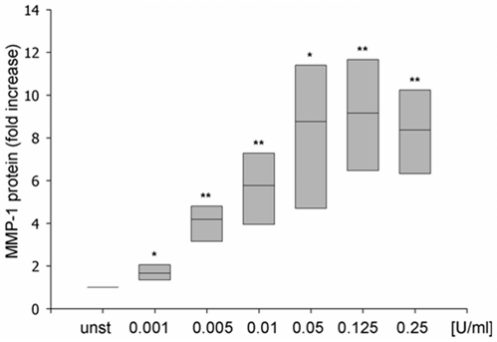
MMP-1 protein expression of Caco-2 IEC is induced by exogenous SMase. Cells were incubated with increasing doses of exogenous SMase. Concentration of active MMP-1 was determined in supernatants after 24h by ELISA. (*p<0.05, **p<0.01; n = 6)

MMP-1 protein production upon generation of ceramide was confirmed by quantification of active MMP-1 by ELISA. Supernatants of Caco-2 cells were analysed 24h after incubation with exogenous SMase (Figure 4). Exogenous SMase led to a dose-dependent increase of MMP-1 with a 3.1-fold increase at concentrations as low as 0.005 U/ml and maximal increase at a concentration of 0.125 U/ml (Figure 4).

### Inhibition of acid sphingomyelinase (ASM) by imipramine completely abrogates the induction of MMP-1 by IL-1βand TNF

**Figure 5:**
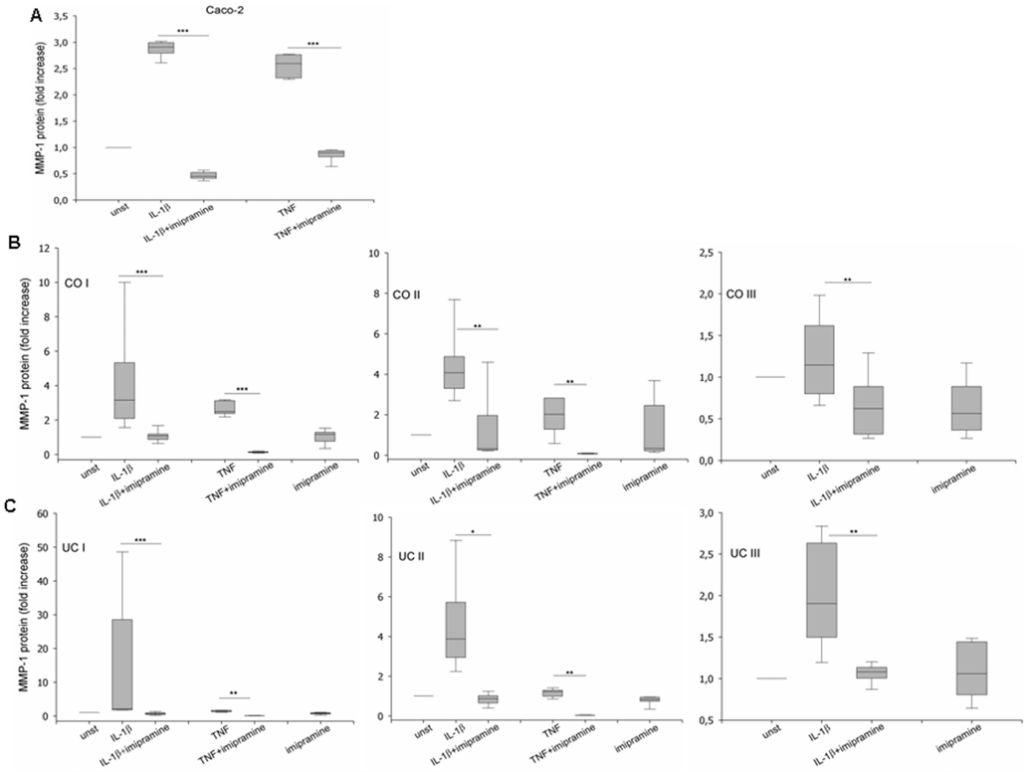
Pharmacological inhibition of ASM by imipramine abrogates induction of MMP-1 by IL-1β and TNF. Concentration of MMP-1 in supernatants was determined after 24h by ELISA. (A) Stimulation of Caco-2 IEC with IL-1β or TNF ± imipramine (n = 5). (B) Stimulation of primary control fibroblasts CO I (n = 12), CO II (n = 9) and CO III (n = 10) with IL-1β or TNF ± imipramine. (C) Stimulation of primary fibroblasts from patients with ulcerative colitis UC I (n = 10), UC II (n = 6) and UC III (n = 6) with IL-1β or TNF ± imipramine. (*p<0.05, **p<0.01; ***p<0.001)

MMP-1 production in Caco-2 cells was induced by stimulation of cells with the ASM-activating inflammatory cytokines TNF (10ng/ml) or IL-1β (1ng/ml). For inhibition of acid sphingomyelinase, cells were incubated with imipramine (30 µM). Imipramine causes proteolytic degradation of ASM [45]–[47] and was therefore added to the cells 30 minutes prior to stimulation. IL-1β induced a 2.9-fold (±0.07) secretion of MMP-1 (Figure 5A) which was completely absent in cells treated with imipramine (Figure 5A). MMP-1 increased by 2.5-fold (±0.1) after stimulation with TNF (Figure 5A) while treatment of the cells with imipramine completely blocked this increase (Figure 5A). These results demonstrate ASM-dependence of IL-1β and TNF for MMP-1 induction in the colon epithelial cell line.

Because fibroblasts essentially contribute to MMP-1 production in IBD, we assessed the occurring changes of MMP-1 in primary intestinal fibroblasts upon stimulation with the ASM-activating inflammatory cytokines TNF and IL-1β. Again, we used imipramine for pharmacologic inhibition of ASM, to analyze the contribution of ASM for production of MMP-1. Induction of MMP-1 by IL-1β or TNF was completely abolished by imipramine in all the investigated primary fibroblasts, including three different sets from healthy control (CO I, CO II, CO III) and three sets from patients with ulcerative colitis (UC I, UC II, UC III) (Figure 5B and 5C). Upon stimulation with IL-1β (1ng/ml) or TNF (10ng/ml), control fibroblasts increased MMP-1 secretion (Figure 5B). Treatment with imipramine abrogated the effect of IL-1β or TNF with significant inhibition when compared to cells with IL-1β or TNF only. Fibroblasts from patients with ulcerative colitis also increased MMP-1 while treatment with imipramine resulted in MMP-1-levels with significant inhibition when compared to cells with IL-1β or TNF only (Figure 5C).

It is important to note that complete serum withdrawal prestimulated the primary fibroblasts with only little response to TNF. Therefore 0.5% FCS was maintained to avoid prestimulation of primary fibroblasts by serum withdrawal.

## Discussion

In the present study, we analysed the levels of bioactive lipids in two models of chronic colitis and identified ceramide as a novel second messenger for disease pathology in IBD. We demonstrate that generation of ceramide and activity of acid sphingomyelinase are required for the production of MMP-1, which is believed to damage the colonic mucosa in patients with ulcerative colitis [21], [22].

Using electrospray ionization tandem mass spectrometry (ESI-MS/MS), we found increased concentrations of ceramide in ex vivo preparations of murine intestinal epithelial cells, and decrease of the degradation product of PC lysophosphatidylcholine. Increased levels of ceramide result from upregulated sphingomyelin breakdown by sphingomyelinases and/or reduced degradation of ceramide by acid ceramidase. To determine the role of the second messenger ceramide, generated by sphingomyelin breakdown, the intestinal epithelial cell line Caco-2 and primary intestinal fibroblasts from patients with ulcerative colitis or healthy controls were challenged with exogenous SMase or the inflammatory cytokines IL-1β and TNF to induce ceramide generation. Affymetrix gene array analysis of Caco-2 IEC revealed dramatic increases of MMP-1 and MMP-10 after generation of ceramide by exogenous SMase. Dose dependent induction of MMP-1 protein expression in Caco-2 IEC upon incubation with exogenous SMase was verified by ELISA. To our knowledge, this is the first description of MMP-1 induction by ceramide in epithelial cells with possible implications for the understanding and treatment of tumor invasion and metastasis. After all, fibroblasts need an inflammatory stimulus in addition to exogenous SMase to induce MMP-1 expression. This regulation is lost in the epithelial cancer cell line Caco-2 which responds with MMP-1 production after generation of ceramide by exogenous SMase alone.

Using acid sphingomyelinase (ASM)-deficient fibroblasts as a genetic model, we have previously shown ASM-dependent induction of MMP-1 by IL-1β [48]. To elucidate the role of sphingomyelin degradation by ASM for MMP-1-induction in IEC and intestinal fibroblasts, activation of ASM was pharmacologically inhibited by imipramine. Imipramine interferes with ceramide signalling, particularly by inhibition of ASM [45]–[47]. TNF and IL-1β, which are known to induce MMP-1 expression as well as to activate ASM, served to evaluate the relevance of ASM-mediated MMP-1-induction [49], [50]. Importantly, inhibition of ASM by imipramine completely abrogated the induction of MMP-1 after stimulation with TNF or IL-1β. These data suggest that generation of ceramide by ASM upon TNF or IL-1β augments intestinal tissue destruction by increase of MMP-1 production which can be prevented by inhibition of ASM. Unfortunately, rodents do not express MMP-1. Therefore, we were not able to explore the effects of ASM-inhibition on MMP-1 expression in our murine models of colitis.

Another consequence of ceramide accumulation may be an increase of cell death and susceptibility to infection, which was demonstrated in a very recent publication by Teichgräber et al, employing an animal model of cystic fibrosis [51]. As shown by Sakata et al., ASM also contributes to increased secretion of inflammatory cytokines in a murine model of experimental colitis [8]. Alteration of membrane fluidity by ceramide has also been shown to affect permeability of IEC [52] and the function of diverse ion channels [53], [54], which may also contribute to extensive loss of fluids and increase of IEC apoptosis in IBD. All of the effects mentioned above may be prevented by inhibition of ASM. Positive effects of imipramine on the clinical course of UC were shown by Esmaeili et al [55]. In view of our results, part of these effects are possibly explained by reduced production of MMP-1.

To address the question of PC administration, which has been reported to be protective in ulcerative colitis [5]–[7], we also compared levels of PC and its degradation product LPC. We did not find direct evidence for a deficiency of PC in intestinal epithelial cells but concentrations of LPC were much lower in inflamed colonic epithelium. This decrease is suggestive of a regulatory response of the mucosa to reduce inflammation, by reduction of inflammatory cytokines like IL-1β [56] and less recruitment of monocytes and T cells [19]. Therefore, the decrease of LPC most likely represents a mechanism of the intestinal mucosa for resolution of inflammation.

In summary, our data demonstrate increased levels of ceramide in experimental colitis. The results indicate a role of ceramide for the pathogenesis of IBD by regulation of MMP-1 expression. Pharmacologic inhibition of ASM by imipramine completely abrogates the induction of MMP-1 upon TNF or IL-1β. Therefore, inhibition of ASM may be a possible treatment strategy for intestinal inflammation, in particular ulcerative colitis.
